# RITA Is Expressed in Trophoblastic Cells and Is Involved in Differentiation Processes of the Placenta

**DOI:** 10.3390/cells8121484

**Published:** 2019-11-21

**Authors:** Julia Maria Wildner, Alexandra Friemel, Lukas Jennewein, Susanne Roth, Andreas Ritter, Cornelia Schüttler, Qi Chen, Frank Louwen, Juping Yuan, Nina-Naomi Kreis

**Affiliations:** 1 Division of Obstetrics and Prenatal Medicine, Department of Gynecology and Obstetrics, University Hospital, Goethe University, Theodor-Stern-Kai 7, D-60590 Frankfurt, Germany; julia-m.wildner@t-online.de (J.M.W.); Alexandra.Friemel@kgu.de (A.F.); Lukas.Jennewein@kgu.de (L.J.); susanne.roth@kgu.de (S.R.); Andreas.Ritter@kgu.de (A.R.); Cornelia.Schuettler@kgu.de (C.S.); louwen@em.uni-frankfurt.de (F.L.); yuan@em.uni-frankfurt.de (J.Y.); 2 Department of Obstetrics and Gynecology, University of Auckland, Auckland 1010, New Zealand; q.chen@auckland.ac.nz

**Keywords:** RITA, preeclampsia, trophoblasts, motility, invasion, fusion

## Abstract

Preeclampsia (PE) remains a leading cause of maternal and perinatal mortality and morbidity worldwide. Its pathogenesis has not been fully elucidated and no causal therapy is currently available. It is of clinical relevance to decipher novel molecular biomarkers. RITA (*R*BP-J (recombination signal binding protein J)-*i*nteracting and *t*ubulin-*a*ssociated protein) has been identified as a negative modulator of the Notch pathway and as a microtubule-associated protein important for cell migration and invasion. In the present work, we have systematically studied RITA’s expression in primary placental tissues from patients with early- and late-onset PE as well as in various trophoblastic cell lines. RITA is expressed in primary placental tissues throughout gestation, especially in proliferative villous cytotrophoblasts, in the terminally differentiated syncytiotrophoblast, and in migrating extravillous trophoblasts. *RITA*’s messenger RNA (mRNA) level is decreased in primary tissue samples from early-onset PE patients. The deficiency of RITA impairs the motility and invasion capacity of trophoblastic cell lines, and compromises the fusion ability of trophoblast-derived choriocarcinoma cells. These data suggest that RITA may play important roles in the development of the placenta and possibly in the pathogenesis of PE.

## 1. Introduction

Preeclampsia (PE) is a pregnancy-specific multi-system hypertensive disorder with a global prevalence of up to 8%, characterized by the de novo onset of concurrent hypertension and proteinuria after 20 weeks of gestation. It is a consequence of diverse pathophysiological processes linked to maternal endothelial dysfunction and systemic inflammation [1,2,3]. PE is subdivided into the early-onset/severe (<34 weeks of gestation) and the late-onset (≥34 weeks of gestation) forms [4]. Early-onset PE mainly originates from the placenta, and is thus also referred to as placental PE. It is tightly associated with defective trophoblast invasion and spiral artery remodeling, while late-onset PE is rather associated with preexisting maternal risk factors like obesity, diabetes, hypertension, or renal disease [5,6]. These classifications have prognostic importance, since placental/early-onset PE entails a greater risk of maternal and fetal complications [7]. Prevention and treatment options are very limited. Therefore, it is still one of the leading causes of maternal and perinatal mortality and morbidity [2].

RITA, the *R*BP-J (recombination signal binding protein J)-*i*nteracting and *t*ubulin-*a*ssociated protein, induces the nuclear export of RBP-J [8], an important transcription factor of the Notch pathway mediated by its residue Thr-143 [9]. RITA has thus been considered as a negative modulator of the Notch pathway [8], which is critically involved in trophoblast differentiation [10]. Additionally, RITA is a microtubule-associated protein (MAP) interfering with tubulin [8,11,12]. We have revealed that, through coating microtubules (MTs), RITA modulates MT stability and dynamics impacting mitotic progression [11,13], and cell migration and invasion [14]. RITA exerts its function in cell motility possibly by influencing several critical cellular events including focal adhesion (FA) dynamics, actin cytoskeleton elasticity, and regulation of MT stability. RITA’s depletion impairs these functions, resulting in reduced FA turnover, migration, and invasion [14]. In support of this notion, enhanced levels of RITA are correlated with unfavorable clinical outcome in anal carcinoma treated with concomitant chemoradiotherapy [15]. However, both overexpression and downregulation of RITA have been shown in various primary tumor entities [15,16], suggesting that its deregulation could influence malignant progression.

In the present work, we have investigated RITA’s function in the placenta and its possible contribution to the pathogenesis of PE. Our data suggest that RITA plays important roles in cell differentiation affecting the invasion and fusion capability of trophoblastic cells.

## 2. Materials and Methods

### 2.1. Tissue Collection

This study was approved by the Ethics Committees at the University Hospital (reference number: 375/11), Goethe University Frankfurt. Written informed approval was obtained from all patients. PE was diagnosed as specified [17]. Tissue samples were taken from each placenta within 30 min post-delivery, fixed in formalin for immunohistochemistry staining (IHC) or frozen immediately in liquid nitrogen for mRNA and protein extraction stored at –80 °C. The six first trimester placenta sections, formalin-fixed and paraffin-embedded, were obtained from the Hospital of Obstetrics and Gynecology of Fudan University, China, as approved by the Ethics Committee at the Hospital of Obstetrics and Gynecology of Fudan University, China (reference number 2018-62). Written consent was obtained from healthy donors undergoing elective surgical termination of pregnancy. Donor age was 20–33 years and gestational age was 6–9 weeks.

### 2.2. RNA Extraction and Real-Time PCR

For the extraction of total RNAs, RNAeasy kits with column DNase digestion were used according to the instructions (QIAGEN, Hilden, Germany). Reverse transcription was carried out with High-Capacity cDNA Reverse Transcription Kit as instructed (Applied Biosystems, Darmstadt, Germany). The probes were obtained from Applied Biosystems (Applied Biosystems, Darmstadt, Germany). A StepOnePlus Real-time PCR System (Applied Biosystems, Darmstadt, Germany) was used to perform Real-time PCR and the data were analyzed with StepOne Software v2.3 (Applied Biosystems, Darmstadt, Germany). For analyzing primary placental tissue the mean value of expression levels of succinate dehydrogenase complex, subunit A (*SDHA*), TATA box-binding protein (*TBP*), and tyrosine 3-monooxygenase/tryptophan 5-monooxygenase activation protein, zeta polypeptide (*YWHAZ*) served as endogenous controls, as these housekeeping genes are known to be stably expressed in placental tissue [18,19]. For cell culture experiments glyceraldehyde 3-phosphate dehydrogenase (*GAPDH*) was used as endogenous control. All results were shown as relative quantification (RQ) [20].

### 2.3. Immunohistochemistry of Placental Tissue

A standard staining procedure with DAKO EnVision^TM^FLEX peroxidase blocking reagent (K8000, DAKO, Hamburg, Germany) was used to stain formalin-fixed, paraffin-embedded placental tissue sections from PE patients and matched healthy controls as stated [15]. A rabbit polyclonal RITA antibody was made and purified by BioGenes (Berlin, Germany) as described [15]. All slides were counterstained with hematoxylin and analyzed using an AxioObserver.Z1 microscope (Zeiss, Göttingen, Germany). RITA expression was evaluated by two independent investigators (J.M.W., A.F.) without knowledge of the diagnosis. The average of the percentage of positive cells was determined. Ten fields per sample were counted, and 200 villous cytotrophoblasts (CTBs), 50 extravillous trophoblasts (EVTs), and 125 decidual cells (DCs) were evaluated per sample. In the case of the syncytiotrophoblast (STB), the positive area per visual field was estimated. Negative controls included samples stained with control immunoglobulin G (IgG) lacking primary antibody as well as RITA antibody neutralized with its corresponding peptide. The slides were further evaluated by the semi-quantitative H-score method, which takes the staining intensity into account. The H-score is determined by adding the results of multiplication of the percentages of positive stained cells with their staining intensity (scored as 0 for no signal, 1 = weak, 2 = moderate and 3 = strong): ((1 × (% cells 1)) + (2 × (% cells 2) )+ (3 × (% cells 3))). The highest possible value is 300 [21,22].

### 2.4. Cell Culture, Transfection, and Treatment

Jar, JEG-3, MDA-MB-231 (ATCC, Wesel, Germany), and BeWo (Sigma-Aldrich, Taufkirchen, Germany) cells were cultured as instructed. The HTR-8/SVneo cell line (referred to as HTR) was kindly provided by Prof. Graham [23] and SGHPL-4 cell line by Professor Whitley [24]. All cell lines were cultured as specified. Small Interfering RNA (siRNA) against the coding region of RITA (GGA AGA AGA ACA AAU ACA G, named siRITA) and siRNA against the 3’-untranslated region (AGG GAA CCC CAG GUA UUA AUU, named siUTR) were obtained from Sigma-Aldrich (Taufkirchen, Germany). Control siRNA was ordered by Qiagen (Hilden, Germany). siRNAs (20 to 30 nM) were transiently transfected with Oligofectamine^TM^ (Thermo Fisher Scientific, Dreieich, Germany) as detailed [25]. GFP-RITA cloning was previously reported [8] and DNA transfection was carried out as described [25]. Trophoblast fusion was induced by treating cells with 25 μM forskolin (FSK, Sigma-Aldrich, Taufkirchen, Germany) for indicated time points. An equal amount of dimethyl sulfoxide (DMSO, Sigma-Aldrich, Taufkirchen, Germany) was used as vehicle control.

### 2.5. Isolation and Purification of Primary Villous Cytotrophoblasts from Human Term Placental Tissue

Villous cytotrophoblast cell isolation and purification was carried out according to Petroff *et al.* [26] and as specified [20,27]. In brief, approximately 50 g of villous placental tissue free of calcification or hematoma were finely minced within 30 min after delivery, rinsed with 0.9% NaCl, and digested with 0.25% trypsin (Thermo Fisher Scientific, Dreieich, Germany) and 300 U/mL DNase I (Sigma-Aldrich, Taufkirchen, Germany) for 20 min, shaking at 37 °C. After digestion, the supernatant was transferred into tubes containing 1.5 mL fetal bovine serum (Merck Millipore, Darmstadt, Germany) and centrifuged (1000× *g*, 15 min). The digestion, transfer, and centrifugation steps were repeated two more times. The pellet was resuspended in DMEM (Thermo Fisher Scientific, Dreieich, Germany) and filtered with a 100-μm cell strainer (Corning, New York, NY, USA). The cells were centrifuged (1000× *g*, 10 min), resuspended in Ca/Mg-free Hank’s balanced salt solution, and stratified on two Percoll gradients (5 to 70%; Sigma-Aldrich, Taufkirchen, Germany). The gradients were centrifuged without brake (1200× *g*, 20 min). The fraction between 35% and 50% of the gradients was used. The fractions were pooled and diluted in pre-warmed medium for centrifugation (1000× *g*, 5 min). The cell pellet was resuspended in medium and the cells were cultured [20,27].

### 2.6. Cell Viability and Invasion Assay

Cell proliferation assays were conducted by using Cell Titer-Blue^®^ Cell Viability Assay (Promega, Mannheim, Germany) as stated [28]. For invasion assay, 24-well transwell matrigel chambers were seeded with cells as described in the manual (Corning, New York, NY, USA) and as previously defined [29,30].

### 2.7. Cell Motility and Time-Lapse Microscopy

Low confluent cells were seeded into 24-well plates and imaged in an environmental chamber. Images were acquired every 5 min for at least 13 hours. All time-lapse images were performed with an AxioObserver.Z1 microscope (Zeiss, Göttingen, Germany) equipped with an AxioCam MRc camera (Zeiss, Göttingen, Germany). Analyses were performed with ImageJ 1.49i software (National Institutes of Health, Bethesda, MD, USA), the manual tracking plugin, and the Chemotaxis and Migration Tool (Ibidi GmbH, Gräfelfing, Germany). All tracks were plotted in GraphPad Prism 7 (GraphPad software Inc., San Diego, CA, USA) and 30 random cells were analyzed per experiment, which was repeated independently three times. The patterns of motility were analyzed; and migration velocity and directionality were calculated as detailed [31].

### 2.8. Western Blot Analysis

Western blot analysis was performed as reported [32]. Cells were harvested with RIPA buffer (50 mM Tris pH 8.0, 150 mM NaCl, 1% NP-40, 0.5% Na-desoxycholate, 0.1% SDS, 1 mM NaF, phosphatase, and protease inhibitor cocktail tablets (Roche, Mannheim, Germany)). The following antibodies were utilized: mouse monoclonal antibody against β-actin (A5441), rabbit polyclonal antibody against human chorionic gonadotropin (β-hCG, SAB4500168) from Sigma-Aldrich (Taufkirchen, Germany), and mouse monoclonal antibody against GAPDH (GTX627408) from GeneTex (Eching, Germany). The RITA antibody was commercially designed and produced (rabbit monoclonal IgG, Epitomics, Burlingame, USA) as stated [11]. The ImageJ 1.48v software (National Institutes of Health, Bethesda, Maryland, USA) was used for densitometry measurements of Western blot analysis.

### 2.9. Immunofluorescence Staining and Measurement

Indirect immunofluorescence was performed as reported [25]. Following primary antibodies were used: rabbit polyclonal antibodies against pan-cadherin (ab16505; Abcam, Cambridge, UK) and against β-hCG (SAB4500168; Sigma-Aldrich, Taufkirchen, Germany), and mouse monoclonal antibody against acetylated α-tubulin (6-11B1, T7451; Sigma-Aldrich, Taufkirchen, Germany). Cy3-conjugated secondary antibodies were obtained from Jackson Immunoresearch (Cambridgeshire, UK). DAPI (4’,6-diamidino-2-phenylindole-dihydrochloride; Roche, Mannheim, Germany) was used to stain the DNA content. All slides were examined with an AxioObserver.Z1 microscope (Zeiss, Göttingen, Germany) equipped with an AxioCam MRm camera (Zeiss, Göttingen, Germany). The immunofluorescence stained slides were further imaged with an confocal laser scanning microscopy (CLSM) as described [30].

### 2.10. Fusion Assay and β-hCG ELISA

Fluorescence microscopy images of pan-cadherin (visualization of cell boundary) and DAPI (DNA) were merged for the quantification of cell fusion as described [27]. Percentages of fused cells were counted and calculated as follows: nuclei inside cell boundaries/total number of nuclei. To quantify the amounts of β-hCG, conditioned medium was harvested and centrifuged (11,270× *g*, 5 min, 4 °C) to remove cellular debris. Measurement of secreted β-hCG was performed with β-hCG enzyme-linked immunosorbent assay (ELISA) according to producer information (EIA-1911, DRG Diagnostics, Marburg, Germany).

### 2.11. Statistical Analysis

Outliers were calculated with Grubbs test (GraphPath QuickCalcs, San Diego, CA). Student’s *t*-test (two-tailed and paired or homoscedastic) was applied to evaluate the significant difference between two conditions with real-time PCR, IHC quantification, cell viability, invasion assay, ELISA, and fusion assay. The statistical evaluation of time-lapse microscopy was performed with an unpaired Mann–Whitney U test (two-tailed). Differences were defined as statistically significant when *p* < 0.05.

## 3. Results

### 3.1. RITA is Specifically Expressed in Placental Tissue and its mRNA Level Decreases at Late Gestational Stages

**Figure 1:**
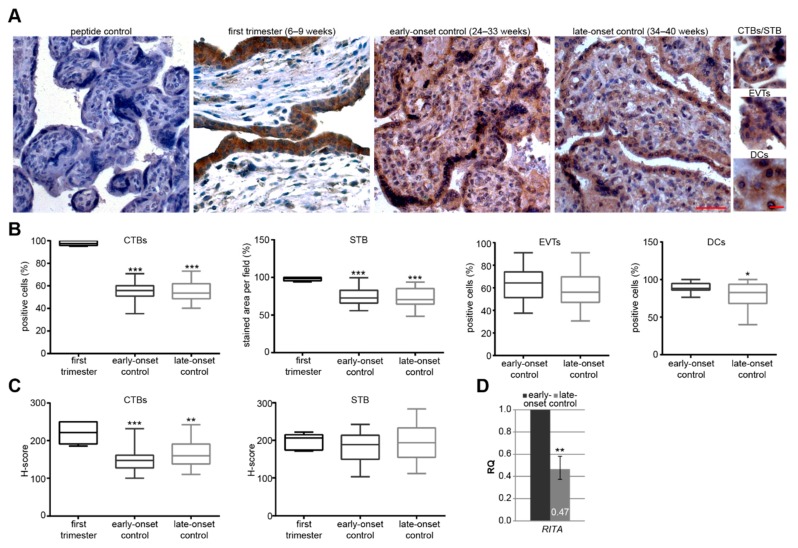
*R*BP-J (recombination signal binding protein J)-*i*nteracting and *t*ubulin-*a*ssociated protein (RITA) is expressed in trophoblastic cells of placental tissue and its gene expression decreases at late stages of gestation. (**A**) Paraffin-embedded tissue sections were immunohistochemically stained with a specific RITA antibody (brown) and counterstained with hematoxylin (blue). RITA antibody neutralized with its corresponding peptide (peptide control) was used as negative control. Scale of images: 50 µm, scale of small images: 20 µm. CTBs, cytotrophoblasts; STB, syncytiotrophoblast; EVTs, extravillous trophoblasts; DCs, decidual cells. (**B**) Quantification of RITA positive cells in first trimester placental sections (6–9 weeks, *n* = 6), early-onset control (24–33 weeks; *n* = 20), and late-onset control samples (34–40 weeks; *n* = 21). The results are presented as box and whisker plots with minimum and maximum variations. Student’s *t*-test, * *p* < 0.05, ** *p* < 0.01, *** *p* < 0.001. (**C**) Semi-quantitative analysis of the RITA staining using the H-score method. The results are presented as box and whisker plots with minimum and maximum variations. Student’s *t*-test referring to first trimester samples, ** *p* < 0.01, *** *p* < 0.001. (**D**) The relative amount of the gene *RITA* was analyzed from placental tissues from late-onset (*n* = 17, 34–40 weeks) compared to early-onset controls (*n* = 13, 26–33 weeks). The results are presented as relative quantification (RQ) with minimum and maximum range and statistically compared between both groups. Student’s *t*-test, ** *p* < 0.01. The mean value of the expression levels of succinate dehydrogenase complex, subunit A (*SDHA*), TATA box-binding protein (*TBP*), and tyrosine 3-monooxygenase/tryptophan 5-monooxygenase activation protein, zeta polypeptide (*YWHAZ*) was used as endogenous control. Clinical information is listed in Table 1.

In order to gain insights into possible roles of RITA in placental development, we obtained first trimester placental tissues derived from healthy donors with gestational ages between 6–9 weeks (*n* = 6). Furthermore, we have collected placental tissues from gestational age, body mass index (BMI) and maternal age-matched donors after birth (clinical information is summarized in Table 1). In parallel to early-onset and late-onset PE, the healthy groups were named early-onset controls (gestational age 24–33 weeks, *n* = 20) or late-onset controls (weeks 34–40 of pregnancy, *n* = 21), respectively. Protein expression of RITA was analyzed in placental tissues of first trimester, early-onset controls and late-onset controls using immunohistochemistry (IHC). Placental sections were stained with a specific RITA antibody [15] and counterstained with hematoxylin. No staining signal was observed in placental tissue stained with RITA antibody neutralized with its corresponding peptide, evidencing that the RITA signal is specific. The positive staining of RITA was predominantly found in the cytoplasm of trophoblastic cells, especially in the proliferative villous cytotrophoblasts (CTB) and the terminally differentiated, non-proliferative, and multinucleated syncytiotrophoblast (STB) throughout gestation (Figure 1A). First trimester sections showed almost 100% positive staining of CTBs and the STB. Unfortunately, there were no extravillous trophoblasts (EVTs) or decidual cells (DCs) detectable in the first trimester placental sections, whereas RITA-positive EVTs and DCs were observable in the placental sections of early- and late-onset controls. Interestingly, there is a significant difference in the percentage of positive CTBs, the positive stained area per field of the STB (Figure 1B), and the H-score of CTBs (Figure 1C) between first trimester sections and early- or late-onset controls, respectively. By contrast, there was no obvious difference in the percentages of positive CTBs or EVTs in the positive stained area per visual field of the STB or in the H-scores between early-onset and late-onset controls. Moreover, DCs, localized in the maternal decidua interacting with EVTs [33], showed a significant reduction in the staining intensity of RITA in placental tissues derived from early-onset relative to late-onset controls. Next, we analyzed the mRNA level of placental tissue samples from early- and late-onset controls using real-time PCR (RT-PCR). The relative amount of the gene *RITA* was reduced by over 50% in late-onset (34–40 weeks, *n* = 17) compared to early-onset control placentas (26–33 weeks, *n* = 13) (Figure 1D).

### 3.2. The mRNA Level of RITA is Decreased in Early-Onset Preeclamptic Placentas

We then compared the expression levels of placental RITA between early- and late-onset PE, and matched healthy control samples of both groups by paying special attention to gestational age, BMI, and maternal age (Table 1). There was no significant difference in gestational age, BMI, and maternal age between early-onset or late-onset PE patients and their controls, respectively.

**Figure 2:**
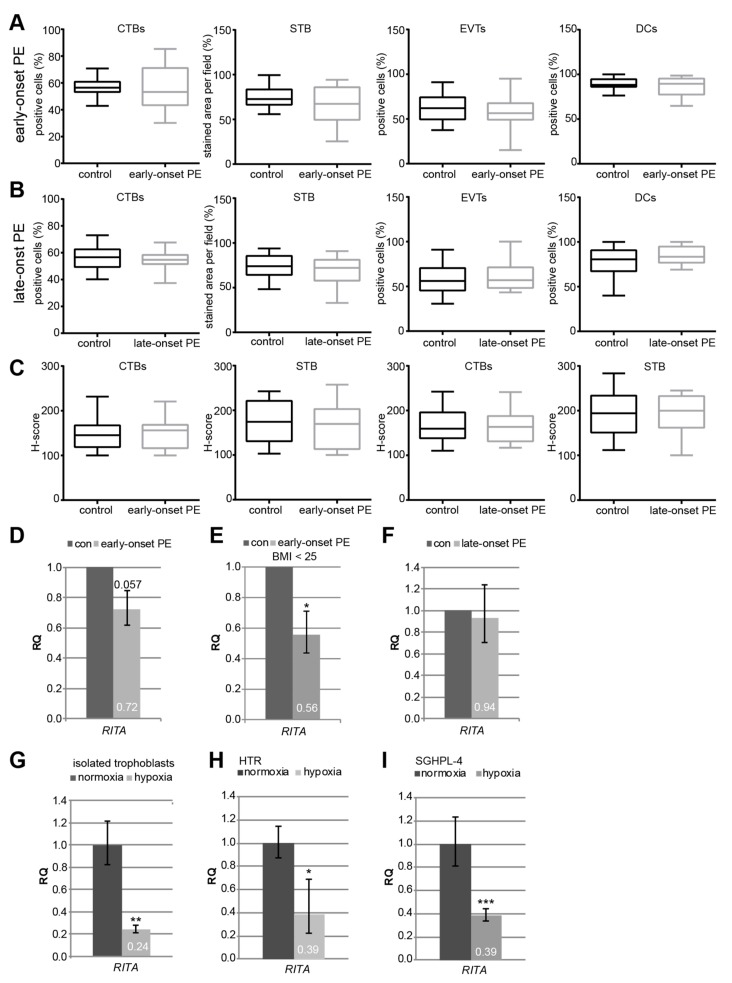
*RITA*’s gene expression is decreased in early-onset preeclampsia (PE) samples, in isolated primary trophoblasts and first trimester-derived cell lines under hypoxic conditions. (**A**–**B**) Immunohistochemical staining of RITA in placental sections was analyzed. Comparison of RITA-positive cells between early-onset PE (25–33 weeks, *n* = 15) and matched controls (24–33 weeks, *n* = 16) (**A**), and between late-onset PE (34–40 weeks, *n* = 14) and matched controls (34–40 weeks, *n* = 19) (**B**). The results are presented as box and whisker plots with minimum and maximum variations. (**C**) Quantification of RITA in CTBs and STB using the H-score method. The results are presented as box and whisker plots with minimum and maximum variations. Clinical information is listed in Table 1. (**D**–**I**) Gene analysis. (**D**) The relative amount of the gene *RITA* was determined in early-onset PE (25–33 weeks, *n* = 14) compared to matched control samples (26–33 weeks, *n* = 13). Student’s *t*-test, *p* = 0.057. (**E**) The relative amount of *RITA* is significantly reduced in placentas derived from patients with a body mass index (BMI) below 25 (*n* = 8) relative to controls (*n* = 6). Student’s *t*-test, * *p* < 0.05. (**F**) The relative amount of placental *RITA* in late-onset PE (34–40 weeks, *n* = 15) compared to the matched controls (34–40 weeks, *n* = 18). The results are presented as relative quantification (RQ) with minimum and maximum range and statistically analyzed between both groups. Clinical information is listed in Table 1. The mean value of the expression levels of three housekeeping genes succinate dehydrogenase complex, subunit A (*SDHA*), tyrosine 3-monooxygenase/tryptophan 5-monooxygenase activation protein, zeta polypeptide (*YWHAZ*), and TATA box-binding protein (*TBP*) was used as endogenous controls. (**G**) Isolated primary cytotrophoblasts were grown under normoxia (21.4% O_2_) and hypoxia (1% O_2_) for 48 h prior to RNA extraction. The gene level of *RITA* was evaluated (*n* = 2, each in duplicates). Glyceraldehyde-3-phosphate dehydrogenase (*GAPDH*) served as housekeeping gene control. Student’s *t*-test, ** *p* < 0.01. (**H**–**I**) Gene analysis of *RITA* depending on the O_2_ level (normoxia, 21.4% O_2_ versus hypoxia, 1% O_2_). HTR (**H**) and SGHPL-4 (**I**) cells were harvested after 48 h. The gene level of *RITA* was measured (*n* = 4, each in triplicates). *GAPDH* was used as housekeeping gene control. Student’s *t*-test, * *p* < 0.05, *** *p* < 0.001.

The staining intensity of RITA’s protein expression was estimated. Compared to their respective healthy counterparts, there was no apparent difference in the percentage of positive CTBs, EVTs, and DCs, or in the positive stained area per visual field in the STB in the early-onset PE (Figure 2A) or the late-onset PE group (Figure 2B). There was also no difference in the intensity by using the H-score method (Figure 2C). By contrast, the relative amount of the gene *RITA* was decreased to 72% in early-onset preeclamptic placentas (early-onset PE, *n* = 14), in comparison to matched control placentas (con, *n* = 13), with a significance of 0.057 (Figure 2D). Excluding patients with a BMI greater than 25, the gene level of placental *RITA* was significantly reduced to 56% between early-onset PE (*n* = 8) and controls (*n* = 6) (Figure 2E), indicating a potential involvement of overweight/obesity in the gene expression of *RITA*. The *RITA* gene level of late-onset PE placentas (late-onset PE, n = 14) was hardly changed compared to controls (con, *n* = 17) (Figure 2F).

PE is associated with constant hypoxia of the placenta causing defective trophoblast invasion and inadequate remodeling of the maternal spiral arteries [1,3,5,34]. To address if the gene expression of *RITA* is affected by low oxygen, primary cytotrophoblasts were isolated from term placentas, which were grown under normal (21.4% O_2_) and hypoxic conditions (1% O_2_). RNA was isolated after 48 h and the gene level of *RITA* was measured. Remarkably, it was significantly reduced to 24% under low oxygen supply (Figure 2G), which specifically mimics the situation of early-onset PE. This was further corroborated with the immortalized normal first trimester trophoblast cell lines HTR-8/SVneo (referred to hereafter as HTR) [23] and SGHPL-4 [24]. Both showed a reduction in *RITA*’s mRNA level upon hypoxia (Figure 2H–J).

### 3.3. Expression and Localization of RITA in Trophoblastic Cell Lines

**Figure 3:**
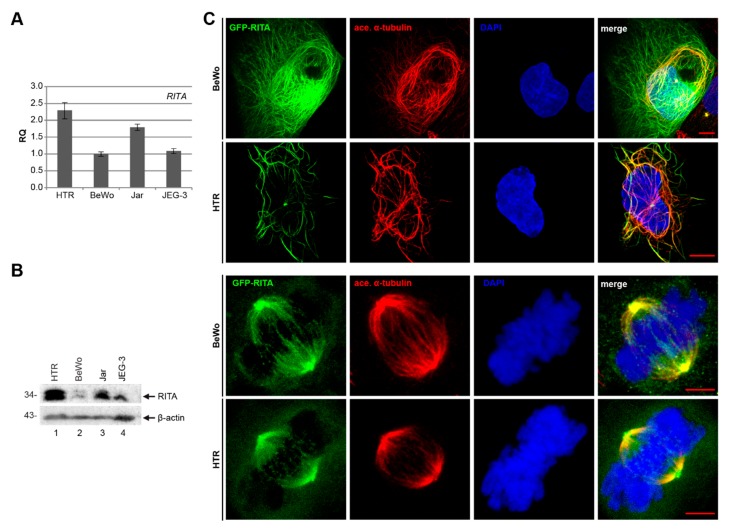
Expression and localization of RITA in various trophoblastic cell lines. (**A**) The relative amount of the gene *RITA* in diverse trophoblastic cell lines. The results are presented as relative quantification (RQ) with minimum and maximum range, by setting the *RITA* value of BeWo cells as 1. (**B**) Western blot analysis with cellular extracts from HTR, BeWo, Jar, and JEG-3 using an antibody against RITA. β-actin served as loading control. (**C**) HTR and BeWo cells were transfected with GFP-RITA and stained for the microtubule marker acetylated α-tubulin and DNA (4’,6-diamidino-2-phenylindole-dihydrochloride (DAPI)). Representatives of the subcellular localization of RITA are shown. First and second row scale: 10 µm, third and fourth row scale: 5 µm.

For a deeper understanding of RITA’s function in placental cells, we examined the mRNA and protein level of RITA in four well-characterized trophoblastic cell lines: BeWo, JEG-3, and Jar, derived from choriocarcinoma, and the first trimester trophoblast cell line HTR. Compared to the other cell lines, the most invasive HTR cell line expresses the highest level of RITA in gene and protein (Figure 3A,B), indicating the possible involvement of RITA in early stages of placental development. The observed double bands of RITA are probably linked to posttranslational modifications of the protein [11]. To examine subcellular localization, cells were transfected with GFP-RITA constructs and stained for the microtubule marker acetylated α-tubulin and DNA followed by confocal microscopy (Figure 3C). GFP-RITA was found to colocalize with cytoplasmic MTs in interphase cells as well as the mitotic spindle as reported for different cancer cell lines and mouse embryonic fibroblasts [8,11,13].

### 3.4. Gene Silencing of RITA Decreases Motility and Invasion Capability of Trophoblast Cells Derived from First Trimester Placentas

**Figure 4:**
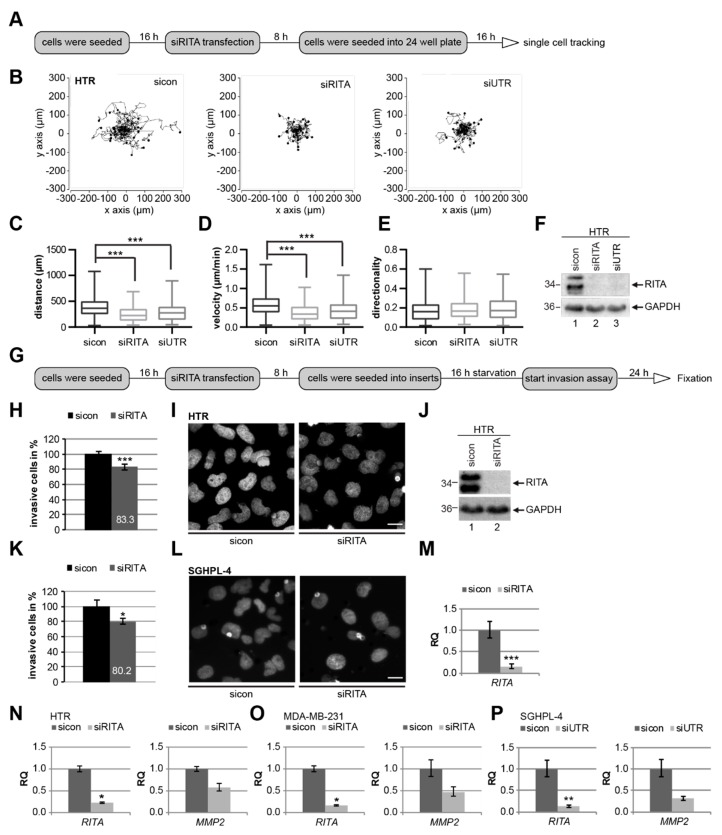
RITA is required for cell motility and invasion of trophoblastic cells. (**A**) Working schedule for time-lapse microscopy. HTR cells were treated with control small interfering RNA (siRNA) (sicon), siRNA against the coding region of RITA (siRITA), or its untranslated region (siUTR) for time-lapse microscopy to analyze their random motility. (**B**) Representative trajectories of individual cells (*n* = 30) are shown. (**C**–**E**) Results of the accumulated distance (**C**), velocity (**D**), and directionality (**E**) are presented as box and whisker plots with minimum and maximum variations. Mann–Whitney U test, *** *p* < 0.001. (**F**) Control Western blot analysis was performed. GAPDH was used as loading control. (**G**) Working schedule for invasion assay. After seeding siRNA-treated cells (sicon or siRITA) into transwell systems, they were starved for 16 h. Then they were released into fresh medium for 24 h and fixated. (**H**) Invasion assay of HTR cells. The total number of invaded sicon cells was assigned as 100%. The results from three individual experiments are presented as mean ± standard error of the mean (SEM). Student’s *t*-test, *** *p* < 0.001. (**I**) Representative images of invaded HTR cells are shown. Scale: 20 μm. (**J**) Control Western blot analysis was performed. GAPDH served as loading control. (**K**) Invasion assay of SGHPL-4 cells. The total number of invaded sicon cells was assigned as 100%. The results from four independent experiments are presented as mean ± SEM. Student’s *t*-test, * *p* < 0.05. (**L**) Representative images of invaded SGHPL-4 cells are shown. Scale: 20 μm. (**M**) Gene analysis served as transfection efficiency control. *GAPDH* was used as housekeeping gene control. The gene expression of *RITA* is presented as relative quantification (RQ) with minimum and maximum range. Student’s *t-*test, *** *p* < 0.001. (**N**) Gene analysis of HTR cells treated with sicon or siRITA. Gene expression of *RITA* or matrix metalloproteinase 2 (*MMP2*) in HTR cells is presented as relative quantification (RQ) with minimum and maximum range. Student’s *t*-test, * *p* < 0.05. (**O**) Gene expression of *RITA* or *MMP2* in MDA-MB-231 cells is presented as relative quantification (RQ) with minimum and maximum range. Student’s *t*-test, * *p* < 0.05. (**P**) Gene analysis of SGHPL-4 cells treated with sicon or siUTR. Gene expression of *RITA* or *MMP2* is presented as relative quantification (RQ) with minimum and maximum range. Student’s *t*-test, ** *p* < 0.01.

Trophoblastic cell migration and their invasion into the maternal decidua will ensure the success of spiral artery remodeling and pregnancy, and failures are often associated with pregnancy-associated disorders [2,3]. RITA’s high expression in EVTs (Figure 1) and HTR cells (Figure 3A,B) suggests its potential function in migration and invasion in early stages of placental development. To address this suggestion, single cell tracking in real-time was performed (Figure 4A). Single HTR cells, treated with control siRNA or two different siRNAs targeting RITA’s coding region (siRITA) or its untranslated region (siUTR) (Figure 4F), were tracked by time-lapse microscopy in order to evaluate their migratory parameters (Figure 4B–E). Whereas the directionality was unaltered (Figure 4E), a significant decrease in the accumulated distance and velocity was observed in RITA knocked down HTR cells compared to control cells (Figure 4C,D). The cell viability was hardly changed (Figure S1A to C). Similar results were also achieved with SGHPL-4 cells (Figure S1D to F).

Since RITA is a negative Notch regulator [9] and PE is associated with defects in trophoblast differentiation critically regulated by the Notch pathway [10], we were interested if the Notch target genes hairy and enhancer of split 1 (*HES1*) and hairy/enhancer-of-split related with YRPW motif protein 1 (*HEY1*) changed their expression upon depletion of RITA in trophoblastic cell lines. However, the expression of both Notch target genes, which are known to be downregulated in PE [35,36], were not influenced by RITA depletion in HTR and SGHPL-4 cells (Figure S1G and H). MDA-MB-231 cells, metastatic breast cancer cells, also showed no alteration in these genes upon the depletion of RITA (Figure S1G and H).

To underline the observations of the reduced motility upon RITA depletion, matrigel-coated transwell invasion assays were performed (Figure 4G), which allow a more direct measurement of the invasive capabilities of trophoblasts [37]. Knockdown of RITA with siRNA reduced the number of HTR cells passing the matrigel by about 17% (Figure 4H–J) compared to corresponding control cells. Consistently, SGHPL-4 cells depleted of RITA showed a decrease in invasion up to 20% (Figure 4K–M). These data provide strong evidence that RITA might be involved in the modulation of cell motility of trophoblastic cells. Since invasion of trophoblasts, like cancer cells, is closely dependent on the expression of matrix metalloproteinases (MMPs), which can degrade the extracellular matrix (ECM) [38], we assumed an alteration in the mRNA level of *MMP2* in cells treated with siRNA against RITA (siRITA). Indeed, the mRNA expression of *MMP2* was reduced in HTR cells (Figure 4N). Further support was obtained from breast cancer cell line MDA-MB-231 (Figure 4O) and trophoblastic SGHPL-4 cells treated with the second siRNA against RITA (siUTR) (Figure 4P). Taken together, our data suggest that depletion of RITA has a negative impact on cell motility and invasion of trophoblastic cells.

### 3.5. Knockdown of RITA Reduces the mRNA Level of Different Fusion-Related Molecules

**Figure 5:**
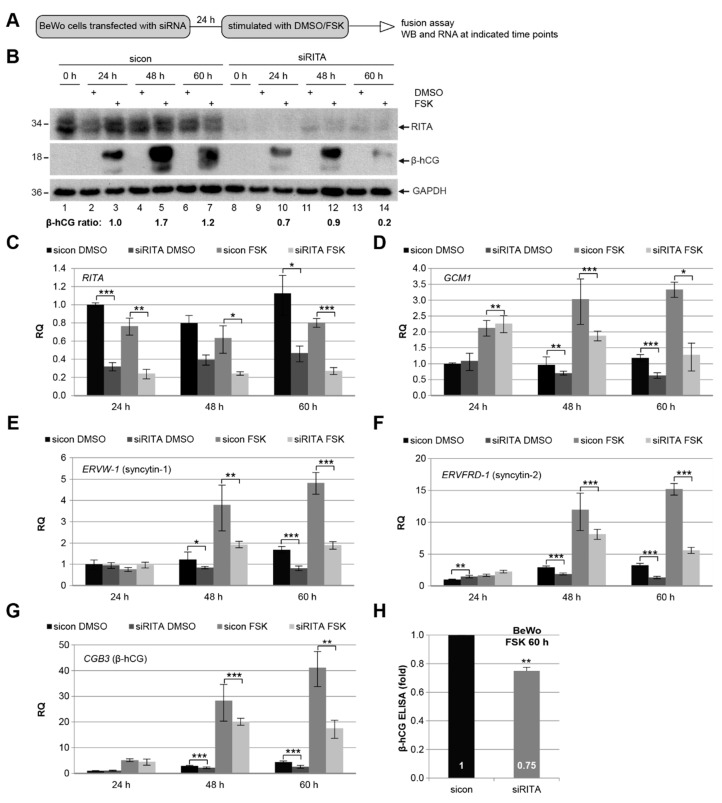
Depletion of RITA leads to a reduction of fusion related genes in BeWo cells. (**A**) Working schedule for fusion assay. BeWo cells were transfected with control siRNA (sicon) or siRNA targeting RITA (siRITA) and then stimulated with forskolin (FSK) to induce cell fusion or vehicle control dimethyl sulfoxide (DMSO) up to 60 h. (**B**) Western blot analysis of treated cells with antibodies against RITA and chorionic gonadotropin subunit beta 3 (β-hCG). GAPDH was used as loading control. The ratios of β-hCG/GAPDH are shown. (**C**–**G**) Gene analysis. The mRNA levels of *RITA* (**C**) and fusion-related glial cells missing transcription factor 1 (*GCM1*) (**D**), syncytin-1 (*ERVW-1*) (**E**), syncytin-2 (*ERVFRD-1*) (**F**) and β-hCG (*CBG3*) (**G**) are shown. The results are presented as relative quantification (RQ) with minimum and maximum range. Student’s *t*-test, * *p* < 0.05, ** *p* < 0.01, *** *p* < 0.001. (**H**) Level of secreted β-hCG. The supernatants of BeWo cells treated with FSK for 60 h were collected for the measurement of secreted β-hCG. The data from three independent experiments are presented as mean ± SEM. Student’s *t*-test, ** *p* < 0.01.

As RITA localizes to CTBs and the STB in the primary placental tissue (Figure 1), we assumed its role during the fusion process. To address this issue, we used the well-established fusogenic choriocarcinoma cell line BeWo, which was stimulated to fuse with forskolin (FSK) up to 60 h (Figure 5A). To find out if RITA impacts the protein and gene expression of fusion-related molecules, cellular lysates as well as total mRNAs were isolated from cells treated with siRITA and FSK. Western blot analysis showed that the intracellular level of chorionic gonadotropin subunit beta 3 (β-hCG), which is up-regulated upon cell fusion and induced by FSK [36], was reduced upon RITA depletion with siRNA. Interestingly, the level of β-hCG was lowered throughout the time course (Figure 5B), with the strongest reduction at 60 h upon stimulation (Figure 5B, second panel, lane 7 and 14). Next, we investigated the gene levels of multiple fusion-related genes including the fusion-specific transcription factor glial cells missing transcription factor 1 (*GCM1*), which has been shown to increase cell fusion of human CTBs [39], and the fusion markers syncytin-1 (encoded by the gene *ERVW-1*), syncytin-2 (*ERVFRD-1*), and β-hCG (*CBG3*) (Figure 5D–G). The analyzed fusion-related genes showed a significant reduction at 48 h and 60 h in BeWo cells depleted of RITA, which were then stimulated with FSK. Intriguingly, reduced mRNA levels of *GCM1*, syncytin-1 (*ERVW-1*), syncytin-2 (*ERVFRD-1*), and β-hCG (*CBG3*) were even observed in the non-fusogenic choriocarcinoma cell line JEG-3 after knockdown of RITA (Figure S2). This indicates that the expression of these genes is generally affected by RITA in trophoblastic cells. To underscore these findings, supernatants were collected for the measurement of secreted β-hCG. Compared to control cells, secreted β-hCG was significantly decreased by 25% in supernatants from cells depleted of RITA and treated with FSK for 60 h (Figure 5H).

**Figure 6:**
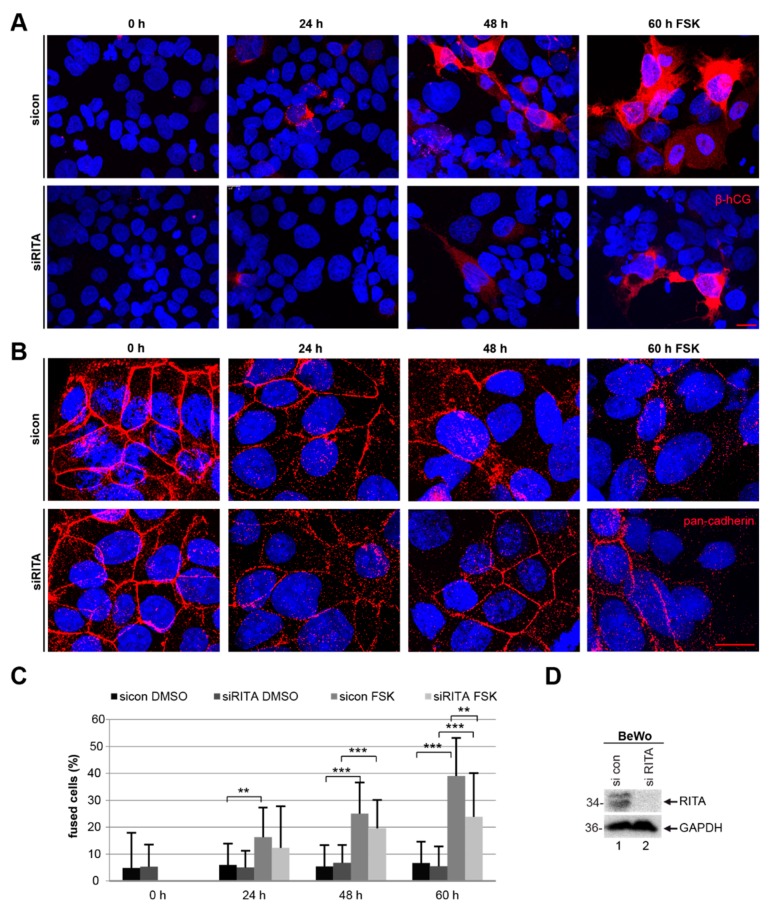
Suppression of RITA impairs the fusion rate of BeWo cells. BeWo cells were treated with control siRNA (sicon) or siRNA targeting RITA (siRITA) for 24 h and additionally with forskolin (FSK) or DMSO for indicated time periods. (**A**) Treated BeWo cells were fixated and stained for the fusion marker β-hCG (red) and DNA (DAPI, blue). Examples are shown. Scale: 25 µm. (**B**) The treated BeWo cells were stained for pan-cadherin (red) and DNA (DAPI, blue). Representative images are shown. Scale: 25 µm. (**C**) For microscopic evaluation and quantification of fused cells, the pan-cadherin (red) and DNA (DAPI, blue) staining were used. The results are displayed in percentages at indicated time periods. The results are shown as mean ± standard deviation (SD). Student’s *t*-test, ** *p* < 0.01, *** *p* < 0.001. (**D**) Western blot analysis using antibodies against RITA and GAPDH. GAPDH served as loading control.

In addition, BeWo cells depleted of RITA showed a reduction in the signal intensity of β-hCG by immunofluorescence staining (Figure 6A, lower panel) compared to control cells (Figure 6A, upper panel). Furthermore, the cells were stained for pan-cadherin, labelling a family of integral membrane proteins, and DNA at indicated time points for microscopic evaluation. The staining revealed a significant reduction in the number of fused cells after RITA depletion (Figure 6B–D). Upon stimulation with FSK for 60 h, knockdown of RITA resulted in 24 ± 16 % cell fusion, compared to 39 ± 14% in cells treated with control siRNA. The data indicate that suppression of RITA attenuates the fusion process of trophoblastic BeWo cells.

## 4. Discussion

In the current study we show that RITA is expressed in CTBs, the STB, EVTs and DCs of primary placental tissue. Moreover, the gene level of placental *RITA* decreases at late gestational stages and it is reduced in early-onset PE. The deficiency of RITA is further associated with impaired motility and invasion capacity of distinct trophoblastic cell lines, and attenuated fusion ability of trophoblast-derived choriocarcinoma cells.

In primary placental tissue, the protein level of RITA is high in first trimester-derived placental sections (6–9 weeks of pregnancy), decreases in the early-onset controls (24–33 weeks), and remains relatively constant in late-onset controls (34– 40 weeks). This is further supported by its elevated level in HTR cells, derived from the first trimester placenta. The mRNA level of *RITA* is high at early-onset controls and decreases at term controls, whereas the protein level is barely changed between both groups. We cannot exclude alterations in RITA’s subcellular localization and posttranslational modifications as shown in cancer cells [11], which may affect its function during gestation. In addition, the mRNA level does not usually predict the protein amount, and significant correlations were only found in one-third of 1066 analyzed gene products [40]. Further investigations are required with specific antibodies against RITA’s post modifications. Nevertheless, these observations indicate its potential involvement in the early development of the placenta.

Invasion of EVTs into the maternal decidua is indispensable for placental embedment and fetal development [19], and aberrant invasion capability of EVTs leads to shallow placentation in PE [3,5]. Intriguingly, in our present work we reveal that the suppression of RITA using siRNA compromises the migration and invasion ability of various trophoblastic cell lines. RITA is a MT-interacting and MT-coating protein [8,11,12,13], whose depletion causes a significant reduction in MT stability as well as in MT dynamics of mitotic cells [11]. It is well established that MT dynamics is important for cell motility by interfering with multiple cellular activities such as focal adhesion (FA) disassembly [41,42]. Indeed, we have shown that reduced expression of RITA leads to decreased cell motility, ascribed to impaired FA turnover and slowed cytoskeletal dynamics in breast cancer cell lines MCF7 and MDA-MB-231, cervical cancer cell line HeLa, and mouse embryonic fibroblasts [14]. In accordance with these data, we show a significantly impaired motility and invasion capacity of trophoblastic cells upon depletion of RITA. Considering that tumor development and placental formation have many common features [19], reduced invasion of trophoblastic cells should result from similar mechanisms as observed in cancer cell lines [14]. In support of our results, depletion of stathmin, an important regulator of MT dynamics, inhibited migration and invasion of BeWo, JEG-3, HTR, and first trimester primary trophoblasts [43]. Furthermore, we show that downregulation of RITA is associated with reduced *MMP2* in trophoblastic HTR and SGHPL-4 cells, and breast cancer cell line MDA-MB-231. MMPs including MMP2 are key enzymes for the invasion process of trophoblastic cells [44,45,46] and are reduced in PE [47,48,49]. These data clearly suggest that RITA plays a role in migration and invasion of trophoblastic cells, which could be of importance in the pathogenesis of PE. In fact, compared to matched control samples, we observed a decline in *RITA*’s mRNA expression in primary samples of early-onset PE. Persistent oxygen insufficiency along with placental oxidative stress are hallmarks of PE and associated with defective trophoblast invasion and inadequate transformation of maternal spiral arteries [1,3,5,34]. We show here that hypoxia significantly affects the transcriptional regulation of *RITA*, leading to a strong decline of its gene expression in diverse cell lines and isolated primary trophoblasts. In sum, these data implicate that reduced expression of RITA might contribute to defective migration and invasion at early stages of gestation associated with the pathogenesis of PE.

Furthermore, RITA’s depletion compromises the fusion ability of the human fusogenic choriocarcinoma cell line BeWo. RITA is a modulator of the stability and dynamics of MTs, an important part of the cytoskeleton interacting actively with the actin cytoskeleton and FA dynamics [8,11,12,13]. Interestingly, there are dramatic alterations in the actin cytoskeleton and the morphology of FAs during BeWo cell fusion after FSK stimulation [50], which have also been observed during differentiation of murine trophoblast giant cells influencing cell motility [51]. An alteration in cytoskeletal proteins may thus affect cell fusion. This is supported by various studies reporting the involvement of the actin cytoskeleton, the tubulin network and/or tubulin-associated proteins in cell fusion [43,50,52,53]. The detyrosination of α-tubulin and relatively low levels of tubulin tyrosine ligase (TTL) [53], which catalyzes the re-addition of a tyrosine residue to tubulin [54], are critical for the fusion of isolated cytotrophoblasts and BeWo cells [53]. This was ascribed to the fact that the detyrosinated α-tubulin facilitated the aggregation of syncytin-2 and connexin 43 at the plasma membrane, which initiated the fusion process [53]. Moreover, knocking down of the MT-remodeling protein stathmin reduced cell fusion of BeWo cells and isolated primary trophoblasts [43]. Interfering with MT dynamics by using the drug colchicine inhibited β-hCG secretion and STB formation [52]. By influencing the cytoskeleton and FA dynamics [11,14], RITA may interfere with the cell fusion process of trophoblastic cells. We assume that RITA is not directly involved in the cyclic adenosine monophosphate (cAMP) signaling pathway, which is induced upon fusion or forskolin stimulation, respectively [55]. Nevertheless, the depletion of RITA is linked to a reduction of the fusion-associated genes *GCM1*, syncytin-1 (*ERVW-1*), and syncytin-2 (*ERVFRD-1*) as well as β-hCG (*CBG3*) and a decrease in cellular and secreted β-hCG in BeWo cells. Since PE is associated with impaired differentiation and fusion of CTBs [27,56], these data strengthen the concept of RITA’s involvement in the development of PE.

## 5. Conclusions

Placental development and tumor progression have much in common. As in tumor cells, RITA is associated with MTs and the suppression of RITA compromises the migration and invasion of trophoblastic cells, and reduces the fusion ability of human fusogenic choriocarcinoma cells. Given that these processes are known to be deregulated in PE, RITA may be involved in placental development and possibly in the pathogenesis of PE. Further work is required to decipher RITA’s impact on the molecular mechanisms within the development of PE.

## Figures and Tables

**Table 1:** Clinical information of preeclampsia (PE) patients and matched controls. Mean value ± standard derivation is shown.

	*n*	Gestational Age (Weeks)	Body Mass Index (BMI)	Age	Birth Weight (g)	Systolic Blood Pressure (mmHg)	Diastolic Blood Pressure (mmHg)	Proteinuria (mg/24 h)
**Control**	21	28.8 ± 2.5	24.8 ± 3.7	29.6 ± 5.5	1295 ± 768	122 ± 11	73 ± 9	n.d.
**Early-Onset PE**	15	29.5 ± 2.7	25.8 ± 6.0	31.5 ± 5.4	1060 ± 328	165 ± 28	101 ± 17	1966 ± 1356
***p*-Value**		0.428	0.582	0.318	0.223	0.00003	0.00001	
**Control**	22	37.9 ± 1.8	24.2 ± 5.9	31.1 ± 3.4	3050 ± 650	124 ± 20	77 ± 17	n.d.
**Late-Onset PE**	16	37.6 ± 1.5	25.1 ± 7.2	31.7 ± 3.6	2253 ± 565	159 ± 24	102 ± 17	3363 ± 6820
***p*-Value**		0.664	0.678	0.621	0.017	0.0001	0.0002	

## Supplementary Materials

The following are available online at https://www.mdpi.com/2073-4409/8/12/1484/s1, Figure S1: Cell viability and Notch target genes are hardly changed upon RITA depletion, Figure S2: Depletion of RITA leads to a reduction of fusion related genes in JEG-3 cells.
