# Supplementary material for: RITA Is Expressed in Trophoblastic Cells and Is Involved in Differentiation Processes of the Placenta
Source: Cells. 2019 Nov 21;8(12):1484. doi: 10.3390/cells8121484 (PMC6953008; doi:10.3390/cells8121484)
Supplement: Supplementary file 1 [file cells-08-01484-s001.pdf]

Supplementary Materials

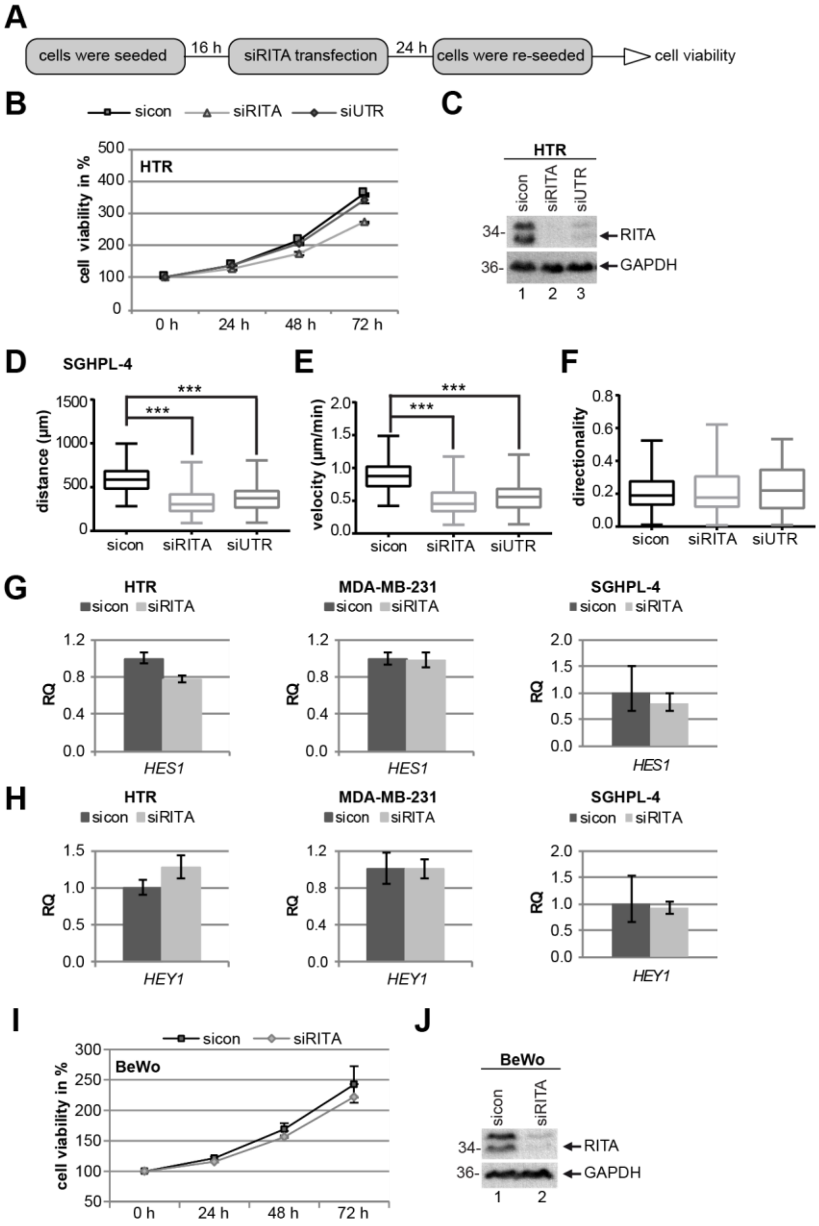

Figure S1. Cell viability and Notch target genes are hardly changed upon RITA depletion. (**A**) Working schedule for cell viability measurement. (**B**) Cell viability of HTR cells transfected with control siRNA (sicon), siRNA against RITA (siRITA) or its untranslated region (siUTR) was measured via CellTiter-Blue® assay. The results from two independent experiments are presented as mean ± standard error of the mean (SEM) and statistically analyzed. (**C**) Cellular lysates were prepared for Western blot analyses with indicated antibodies as transfection efficiency control. GAPDH was used as loading control. (**D-F**) Measurements of the accumulated distance (**D**), velocity (**E**) and directionality (**F**) of SGHPL-4 cells are shown as box plots with variations. Mann-Whitney U test, ***$p < 0.001$. Transfection controls are shown in Figure 4M and P, respectively (**G-H**). Gene expression levels of two Notch target genes hairy and enhancer of split 1 (*HES1*) (**G**), and hairy/enhancer-of-split related with YRPW motif protein 1 (*HEY1*) (**H**) are presented as relative quantification (RQ) with minimum and maximum range in HTR (left panel, n = 3), MDA-MB-231 (middle panel, n = 3) and SGHPL-4 cells (right panel, n = 1). (**I**) Cell viability of BeWo cells transfected with sicon or siRITA was measured via CellTiter-Blue® assay. The results from four independent experiments are presented as mean ± SEM and statistically analyzed. (**J**) Cellular lysates were prepared for Western blot analyses with indicated antibodies as transfection efficiency control. GAPDH served as loading control.

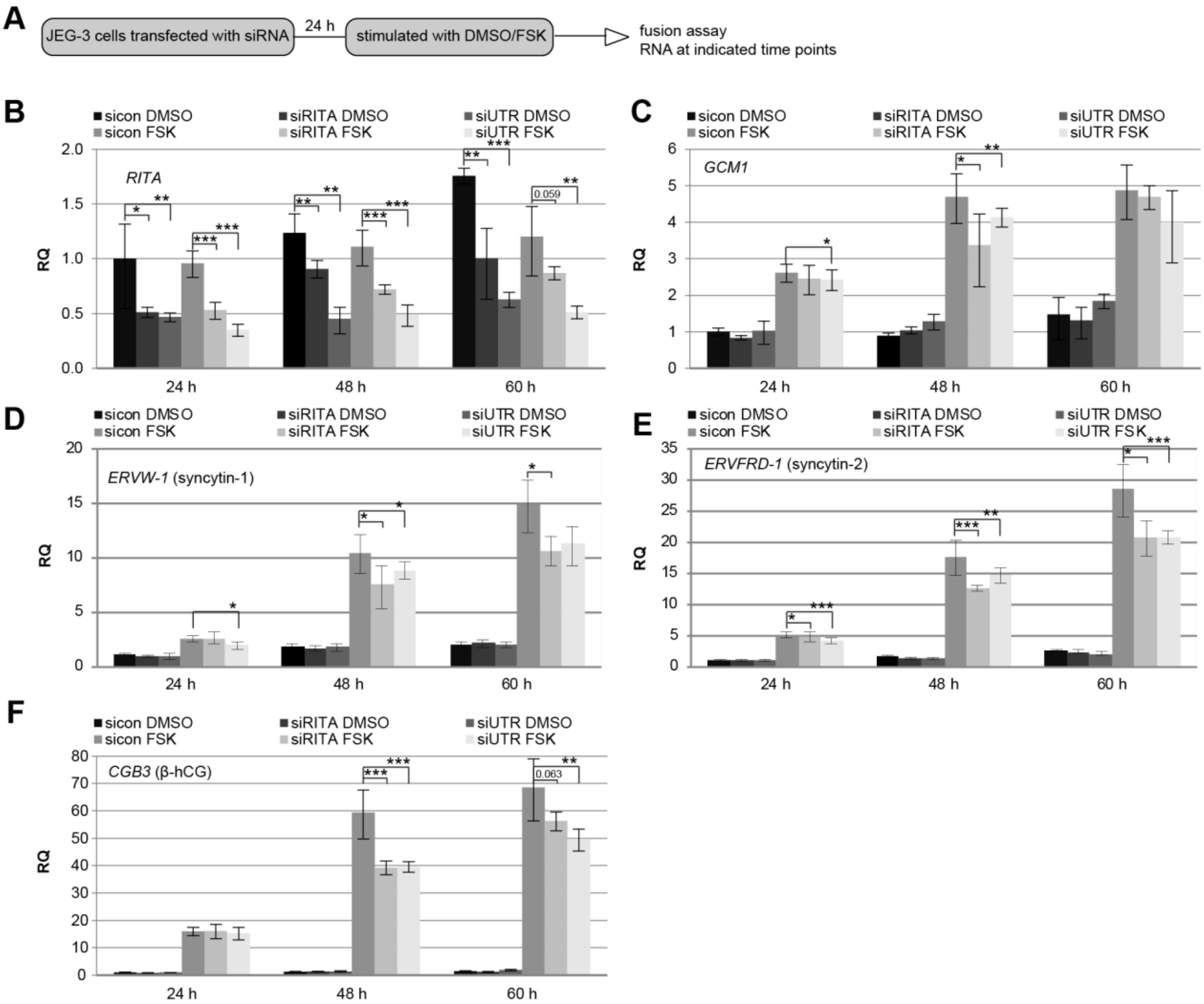

Figure S2: Depletion of RITA leads to a reduction of fusion related genes in JEG-3 cells. (**A**) Working schedule for fusion assay. JEG-3 cells were transfected with control siRNA (sicon) or siRNA targeting RITA (siRITA) or the untranslated region of RITA (siUTR) and then stimulated with forskolin (FSK) to induce cell fusion or treated with dimethyl sulfoxide (DMSO) as vehicle control for indicated time periods. (**B-F**) Total RNA was isolated from treated cells for evaluation of relative amounts of fusion related genes. The mRNA levels of *RITA* (**B**) and fusion related proteins glial cells missing transcription factor 1 (*GCM1*) (**C**), syncytin-1 (*ERVW-1*) (**D**), syncytin-2 (*ERVFRD-1*) (**E**) and chorionic gonadotropin subunit beta 3 (β-hCG, *CBG3*) (**F**) are shown. The results are presented as relative quantification (RQ) with minimum and maximum range. Student's t-test, *p< 0.05, **p< 0.01, ***p< 0.001.
